# Bilateral vestibulopathy affects spatial and temporal perception

**DOI:** 10.1371/journal.pone.0336108

**Published:** 2025-11-06

**Authors:** Olga Kuldavletova, Deborah Cecilia Navarro Morales, Timothy R. Macaulay, Scott J. Wood, Michel Toupet, Charlotte Hautefort, Christian Van Nechel, Anthony Rengel, Adéla Kola, Thomas Fréret, Gaëlle Quarck, Pierre Denise, Gilles Clément

**Affiliations:** 1 COMETE U1075, Université de Caen Normandie, INSERM, CYCERON, Caen, France; 2 Neuroscience Laboratory, NASA Johnson Space Center, Houston, Texas, United States of America; 3 Centre d’Explorations Fonctionnelles Oto-Neurologiques, Paris, France; 4 Université de Paris Cité, INSERM U1141, Paris, France; 5 Department of Otorhinolaryngology, Assistance Publique, Hôpitaux de Paris, Lariboisière Hospital, Paris, France; 6 Australian Antarctic Division, Kingston, Tasmania, Australia; 7 Department of Neurology, CHU de Caen, Caen, France; 8 KBR, Houston, Texas, United States of America; Universiti Malaya Fakulti Perubatan: University of Malaya Faculty of Medicine, MALAYSIA

## Abstract

This study assessed impairments in spatial and temporal perception in individuals with bilateral vestibulopathy (BVP). A total of 30 BVP subjects and 35 healthy controls (CTL) participated in a series of tests to assess their perception of distance (1–6 meters), angle (90–360 degrees), duration (2–10 seconds), and a combination of distance and angle during a triangle completion task (TCT). When performing distance and angle perception tasks separately, the BVP subjects showed larger errors than the CTL subjects. During the TCT, the BVP subjects walked longer paths and exhibited greater angle deviations compared to the CTL subjects. The angle deviations of the BVP subjects during the TCT were larger than when the angle perception task was performed separately. Moreover, the BVP subjects demonstrated accurate time interval perception, whereas the CTL subjects did not. Although the vestibular system is crucial for balance and spatial awareness, the proprioceptive system, in combination with visual and cognitive strategies, as well as motor efference copies, can help individuals with labyrinthine defects in separately perceiving distances and angles. However, this compensatory approach becomes less effective when these tasks are combined. These findings are relevant for space (planetary) exploration because exposure to microgravity mimics loss of vestibular otolith function.

## Introduction

The perception of egocentric distance refers to the perceived distance between an observer and an object. This spatial perception plays a crucial role in everyday activities, such as reaching for a glass, walking through a crowded street, driving a car, or landing an aircraft. Path integration is another critical cognitive skill that allows humans to navigate by continuously updating the distance and the direction they travel from a starting point [1]. This path integration process relies on multiple self-motion cues, including inputs from the visual, vestibular, and proprioceptive systems, as well as motor efference copies [2]. The visual system processes movement to create optic flow, which helps with avoiding obstacles and combines with vestibular signals to determine object locations. The proprioceptive system delivers spatial feedback from physical contact with the environment and helps spatial orientation during movement by mapping limb speed to the body’s internal model [3]. Meanwhile, the vestibular system detects head movements, and distinguishes between linear accelerations (via the saccule and utricle) and angular accelerations (via the semicircular canals) of the head. Signals from the vestibular system feed into head direction cells and place cells to help determine both the direction of the body movement and the location of objects [4].

The adaptation of vestibular function to microgravity significantly affects astronauts’ spatial and temporal perception, leading to challenges in navigation, orientation, and time estimation. Research, including studies from space missions, parabolic flight simulations, and analog environments [5–10], has shown that the absence of gravitational cues causes spatial disorientation and impairs temporal judgment, disrupting tasks that rely on precise sensory integration. Understanding these effects is crucial for developing countermeasures, such as sensory substitution devices, and vestibular and proprioceptive training, to help astronauts adapt to the unique challenges of long-duration space missions and ensure their safety and performance in space.

Bilateral vestibulopathy (BVP) offers a valuable clinical model for studying spatial orientation in the absence of normal vestibular input. It provides insight into how the brain adapts to disrupted vestibular signals—adaptations that closely parallel those required during spaceflight, where microgravity alters vestibular cues. As such, BVP serves as a useful analog for understanding the neural and behavioral adjustments needed during and after spaceflight, with implications for astronaut training and recovery.

Early research determined that individuals with vestibular deficits were unable to accurately judge their displacement after passive whole-body rotations [11]. However, when walking blindfolded towards a previously seen target, no significant difference in distance error was observed between normal and vestibular-impaired groups [12,13]. During tasks requiring more complex movements, such as walking a triangular path, the overall path walked was similar between groups, but vestibular-impaired subjects had difficulty turning at corners [14,15]. The lack of difference, which may seem surprising given that vestibular-deficient patients have significantly higher thresholds for perceiving passive translations and rotations compared to control subjects [16], could be explained by three factors. First, patients with vestibular disorders often compensate for their impairment by using sensory inputs from the visual and somatosensory systems, as well as employing adaptive strategies [17]. This compensation would help sustain the performance of the path integration process, which relies on multisensory, not solely vestibular, information [2]. Secondly, vestibular impairments in earlier studies were not well characterized, partly because diagnostic tools such as video head impulse testing and vestibular evoked myogenic potentials did not yet exist, and could involve variable parts of the vestibular system (semicircular canals and/or otolith), one or both vestibules, and could even involve the central nervous system. Lastly, these studies had relatively small sample sizes (N < 10).

In the present study, we evaluated 30 BVP patients with documented impairment of semicircular canal and otolith function using the diagnostic criteria of the Bárány Society [18]. BVP is a chronic vestibular syndrome characterized by unsteadiness when walking or standing, which worsens in darkness, on uneven surfaces, or during head movement. To further explore the role of vestibular organs in perception of active self-motion, we assessed how BVP patients perceived distance and angle during 2 distinct tasks: walking blindfolded towards a previously seen target; and rotating their body in yaw at various angles with their eyes closed. Additionally, BVP patients performed a triangle completion task (TCT), which involved both active body rotation and translation. Since self-motion perception involves both spatial and temporal encoding [19], and the vestibular system influences time perception [20], we also evaluated how BVP patients estimated durations ranging from 2 to 10 seconds, which corresponds to the timeframes required for these tasks.

The study aimed to evaluate how BVP patients perceive distance, angle, and duration during self-motion tasks, including walking blindfolded toward a target, rotating their body in yaw with eyes closed, and performing a TCT involving both body rotation and translation, and during a task of time reproduction. The performance of the BVP patients was compared to that of a control group of healthy control subjects (CTL). The study assumed that the vestibular system plays a critical role in spatial and temporal perception, and that BVP patients, due to vestibular dysfunction, would show impairments in these tasks, though they might compensate by relying on other sensory systems or adaptive strategies.

## Methods

### Participants

Thirty BVP subjects (13 males, 17 females; 60.6 ± 13.0 years) were recruited from the *Association Française de Vestibulopathie Bilatérale* [21] and tested in the COMETE Laboratory at the University of Caen from 3 October 2022–18 January 2024. Thirty-five healthy control subjects (CTL) (16 males, 19 females; 40.9 ± 10.4 years) participated in a control study in the COMETE Laboratory or in the Neuroscience Laboratories at NASA Johnson Space Center in Houston, Texas from 3 October 2022 to 10 June 2024. This study was approved by the French Ethical Committee (*Comité de Protection des Personnes de la Région Ouest I*, No: ID-RCB 2022-AO1513–40) and the NASA Johnson Space Center Institutional Review Board (STUDY00000325). All subjects provided written informed consent before participating in the study. The test procedures were performed in accordance with the ethical standards laid down in the 1964 Declaration of Helsinki.

The diagnostic criteria for the BVP subjects strictly followed the guidelines outlined in the consensus document by the *Classification Committee of the Bárány Society* [18]. In addition, we excluded patients with hearing loss or neurological symptoms, whether central or peripheral. The BVP subjects in this study experienced the condition for an average of 8 ± 2 years. The caloric nystagmus velocities of all subjects were below 6°/s. Most of the BVP subjects (90%) showed deficits in the video head impulse test, with no compensatory eye movements and multiple catch-up saccades. Twenty-three of the BVP subjects had reduced utricular responses (oVEMP amplitude below 100 µV), although 16 of these subjects retained cVEMP responses, indicating preserved saccular function. None of the BVP subjects experienced positional vertigo or exhibited signs of cerebellar ataxia, although 4 subjects reported oscillopsia, and 5 had frequent migraines. An etiology of BVP was found in 7 patients (ototoxic drugs for 4 patients and genetic causes for 3 patients), the others being considered idiopathic and degenerative in nature, which is in line with the literature given the exclusion criteria we used [22].

### Experimental protocol

The BVP and CTL subjects completed 4 tasks in random order using action-based methods [23]: (a) a distance perception task with blind walking, (b) an angle production task with blind turning, (c) a TCT performed with the eyes closed, and (d) a duration reproduction task. While performing all these tasks, the subjects wore external noise-cancelling earphones that masked any auditory clues of spatial orientation. Due to time constraints, one BVP subject and 4 CTL subjects were unable to participate in some tasks.

### Perception of walking distance

**Fig 1:**
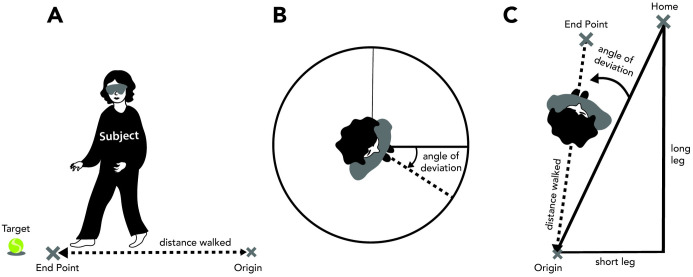
Methods used to assess perception of distance (blind walking) (A), angle (blind turning) (B), and path integration (triangle completion task) (C).

In this task, an operator positioned a target (a tennis ball) at distances of 1, 2, 3, 4, 5, or 6 meters from the subjects, in a random order. One trial was conducted for each distance. The subjects observed the target, and when ready, walked with their eyes closed toward where they believed the target to be [24]. As they walked, the operator quietly removed the target to avoid any contact between the subject and the target that would alert them to the location. After reaching where they perceived the target to be, the subjects, still with their eyes closed, were led back to the starting point so they were unaware of any errors in their perception of distance. The distance from the starting point to the final position of the walk (distance walked) was recorded (Fig 1A). The endpoint error was then calculated as the difference between the ideal distance and the actual distance walked. Because subjects with labyrinthine defects struggle to maintain stable balance while walking without vision [25], 2 operators walked alongside the BVP subjects for support, occasionally holding them to prevent loss of balance or falls. As a result, trajectory deviations were not recorded.

### Perception of self-rotation angle

Participants were instructed to rotate their entire bodies in the yaw plane by 90°, 180°, 270°, or 360° to either the right or left, while keeping their eyes closed (Fig 1B). Two operators were present to ensure the safety of BVP participants and prevent falls during the task. Each participant performed one trial for every angle in both directions, presented in a randomized order. After each trial, a third operator measured the precise angle of body rotation using a protractor. Participants were not realigned between trials and kept their eyes closed throughout, preventing them from receiving any feedback on their performance.

### Triangle completion task (TCT)

The TCT involves blindfolded participants being guided along 2 sides of a triangle before they independently rotate and navigate back to the starting point by relying on their spatial memory and egocentric navigation strategies [26]. This task assesses spatial navigation by characterizing the endpoint errors and angular errors, which reflect the vestibular system’s sensitivity to linear and rotational motion, respectively. In this study, subjects began at a “home” location, moved along 2 fixed legs (short, 92.5 cm, and long, 185.5 cm), and were then asked to return to the home location by walking the shortest route back, forming the third leg of the triangle (207 cm) (Fig 1C). Each subject completed 2 trials: one starting with the long leg and the other with the short leg. They first practiced with their eyes open and then repeated the task with their eyes closed. With the eyes closed, operators guided the subjects through the first 2 legs, after which the subjects independently turned and walked the third leg. Two operators accompanied the BVP subjects for safety. Because the subjects walked only short distances, the operators rarely needed to assist the BVP subjects to prevent loss of balance or falls. The measured variables included the distance walked and the angle of deviation from the home trajectory.

Thomson [24] showed that if subjects walked with their eyes closed for 8 seconds or less, they were able to accurately perceive egocentric distance within a range of 21 meters. However, when the subjects walked blind for more than 8 seconds, the accuracy of their perception of distance decreased rapidly. Consequently, the subjects in our study were instructed to perform the blind walking, blind turning, and TCT within 8 seconds.

### Perception of duration

During the test, seated subjects wore a head-mounted display (Oculus Rift, Oculus VR, Menlo Park, CA), and used a finger trackball connected to a laptop to report their responses [5]. The subjects evaluated the duration of time a blue square was displayed in the center of the screen. At the beginning of each trial, the sentence “Evaluate the target duration” appeared at the bottom of the screen. Then, the square was displayed for a duration of either 2, 3, 5, 7, or 10 seconds (encoding phase). The time intervals (2–10 seconds) were selected because they align with the duration required to complete the distance, angle, and triangle completion tasks.

After the encoding phase, the sentence “Reproduce the duration just evaluated” was displayed on the screen and was pronounced aloud by the computer. The square then reappeared and the subjects pressed the trackball to erase it when they judged that the previously displayed duration was over (reproduction phase). One trial was conducted for each duration.

During both the encoding and reproduction phases, one-digit numbers were displayed in random order in the center of the blue square, with a random inter-digit interval between 350 and 950 milliseconds. Subjects were asked to read aloud these digits. The reproduction response in these dual-task conditions require simultaneously evaluating the duration the target is displayed and performing the concurrent reading task [27]. Therefore, attention is divided between the 2 types of information processing, which impairs the performance of the task. We hypothesized that these impairments would be greater in BVP subjects because they have reduced attention resources.

Duration, distance, angle perception, and triangle completion tasks are crucial for understanding deficits in spatial and temporal integration because they require the coordination of sensory and cognitive processes. Duration perception impacts the timing of movements, while distance and angle perception are essential for navigating space and understanding movement. The triangle completion task combines these elements, requiring both distance and rotation integration to return to the starting point. These tasks highlight how vestibular dysfunction can disrupt the ability to accurately process and combine spatial and temporal information, leading to challenges in navigation and movement coordination.

### Statistical analysis

The differences (errors) between the subjects’ judgments and the actual distance, angle, or durations were calculated for each trial. Relative errors were expressed as a percentage of the actual distance, angle, or duration. For the TCT, the absolute distance and angle errors from the walked path and angle of deviation, respectively, were averaged across both trials.

Data are presented as mean ± standard deviation (SD). Normality was assessed using the Shapiro-Wilk test, while variance homogeneity was evaluated with Levene’s test. Equality of variances was violated for the distance errors, and both normality and equality of variances were violated for angle errors.

Angle and distance errors from the TCT were compared between groups using Welch’s non-parametric t-test. This test was also applied to assess differences between the two experimental groups (CTL, BVP) for each target distance, angle, and duration during the blind walking, blind turning, and duration reproduction tests, respectively. P-values were adjusted for multiple comparisons using the false discovery rate method. Statistical analyses were performed in R with the *stats* and *rstatix* packages, using a two-sided significance level of 0.05 [28].

The residuals of the classical linear mixed-effects model were not normally distributed in the analysis of perception errors during the blind walking, blind turning, and duration reproduction tests. To address this, robust linear mixed-effects models were utilized to investigate the relationships between perception errors (blind walking, blind turning, duration reproduction), subject groups (CTL, BVP), and targets (distance, angle, duration). These models accounted for subject variability to adjust for repeated measurements. The analysis was performed in R using the *robustlmer* function from the *robustlmm* package, which reduces the influence of outliers on both fixed and random effects [28]. P-values were approximated with the *sjPlot* package, which calculated them based on the degrees of freedom from a non-robust mixed linear model.

## Results

### Perception of distance

**Fig 2:**
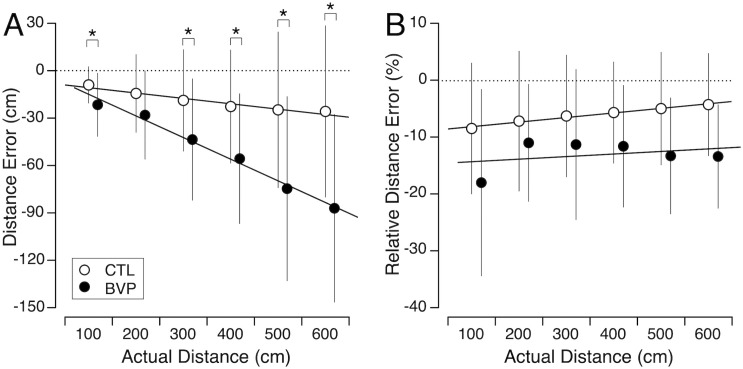
Perception of distance. Mean and standard deviation of distance errors (A) and relative distance errors (B) when judging distance during blind walking towards 6 target distances in a group of healthy control subjects (CTL; N = 31) and a group of bilateral vestibulopathy patients (BVP; N = 29). * *p* < 0.05.

During the blind walking test, CTL subjects consistently stopped short of the target, and their error increased as the target distance increased (Fig 2). The mean relative error, expressed as a percentage of the actual distance, was −5.0%, ranging from −9.0% for the 1-m distance to −4.3% for the 6-m distance. Similarly, the BVP subjects also stopped short, with a mean relative error of −13.2% (ranging from −18.1% to −11.1%). Welch’s test revealed that BVP subjects exhibited significantly greater errors than CTL subjects when judging target distances of 1, 3, 4, 5, and 6 meters (Table 1). The CTL subjects’ absolute trajectory deviation was 1.7 ± 1.2 degrees (mean ± SD). As indicated in the Methods section, the BVP subjects’ trajectory deviations could not be measured.

**Table 1:** Between group comparisons of errors in producing distance, angle, and duration during the blind walking, blind turning, duration reproduction tasks, and triangle completion task, respectively, in the CTL and BVP groups. Comparisons were made using Welch’s non-parametric t-test, and the *p*-value was adjusted for multiple comparisons using the false discovery rate method. * *p* < 0.05.

Measured errors	t	df	Cohen’s d	*p-*value
Distance perception				
1 m	2.851	46.552	0.727	0.012*
2 m	1.756	52.315	0.493	0.075
3 m	1.802	53.998	0.68	0.016*
4 m	−0.255	57.187	0.836	0.006*
5 m	2.882	55.702	0.911	0.004*
6 m	4.083	55.513	1.07	0.001*
Angle perception				
90 deg	−3.445	49.82	−0.905	0.004*
180 deg	−1.239	57.604	−0.32	0.25
270 deg	0.892	54.777	0.231	0.376
360 deg	1.043	54.929	0.27	0.32
Duration perception				
2 s	2.855	44.537	0.753	0.012*
3 s	3.023	35.505	0.813	0.010*
5 s	3.181	42.072	0.829	0.007*
7 s	3.686	39.389	0.962	0.004*
10 s	2.831	43.859	0.746	0.012*
Triangle completion				
distance	−2.245	37.451	−0.541	0.001*
angle	−4.648	34.493	−1.14	0.040*

The robust linear mixed model revealed a significant effect of target distance on judgment error (Table 2). Keeping the distance constant (at the baseline), the model showed no significant difference between the subject groups. However, a significant interaction between target distance and group indicated that the effect of target distance on judgment error was more pronounced in CTL subjects compared to BVP subjects.

**Table 2:** Robust linear mixed model comparing the differences in distance judgement error during the blind walking test between the CTL and BVL subject groups. For the fixed effects, the robust estimation down-weighted 18% of the residuals. For the random effects, the robust estimation down-weighted 13% of the residuals; the analysis included 359 observations in 60 subjects. * *p* < 0.05.

Fixed effects	Distance Error			
Predictors	Estimates	Confidence Interval	*t*-value	*p*-value
(Intercept)	−5.55	−17.41 to 6.31	−0.92	0.358
Distance	−0.04	−0.06 to −0.01	−3.04	0.003*
Group [BVP]	3.82	−13.24 to −20.88	0.44	0.66
Distance x Group [BVP]	−0.09	−0.13 to −0.06	−5.06	< 0.001*
**Random effects**	Variance	SD		
Subject (intercept)	338.3	18.39		
Residual	846.1	29.09		

### Perception of self-rotation angle

**Fig 3:**
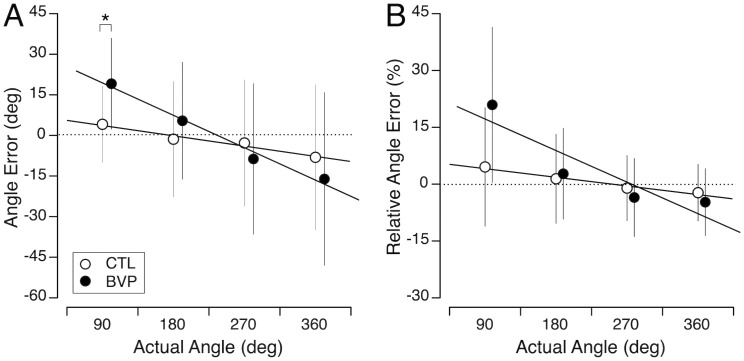
Perception of angle. Mean and standard deviation of angle errors (A) and relative angle errors (B) when judging angle during blind turning for the 4 target angles in the CTL (N = 31) and BVP (N = 29) groups. * *p* < 0.05.

The CTL subjects’ performance during the blind turning test, was highly accurate, with a mean angle error of −2.1 degrees (ranging from 4.1 degrees to −8.1 degrees) (Fig 3). The mean relative error was 0.7%, ranging from 4.5% for the 90-degree angle to −2.3% for the 360-degree angle. In contrast, the BVP subjects over-rotated by 19.1 degrees when attempting to turn 90 degrees, accurately rotated to 180 degrees (mean error was 5.4 degrees), and under-rotated when attempting to turn 270 and 360 degrees (−8.8 degrees and −16.1 degrees, respectively. The mean relative error for the BVP subjects was 4.1% (ranging from 21.1% to −4.5%). Welch’s test revealed that the BVP subjects’ angle error when attempting the 90-degree turn angle was significantly greater than the CTL subjects’ error (Table 1).

The robust linear mixed model revealed a significant effect of target angle on judgment error (Table 3). Keeping the angle constant (at the baseline), the model showed a significant difference between the subject groups. The significant interaction between target distance and group indicated that the effect of target angle on judgment error was more pronounced in BVP subjects than in CTL subjects.

**Table 3:** Robust linear mixed model comparing the differences in angle judgement error during the blind turning test between the CTL and BVL subject groups. For the fixed effects, the robust estimation down-weighted 17% of the residuals. For the random effects, the robust estimation down-weighted 12% of the residuals; the analysis included 239 observations in 60 subjects. * *p* < 0.05.

Fixed effects	Distance Error			
Predictors	Estimates	Confidence Interval	*t*-value	*p*-value
(Intercept)	8.01	−1.15 to 17.18	1.72	0.086
Angle	−0.05	−0.08 to −0.02	−3.12	0.002*
Group [BVP]	21.47	−8.22 to 34.71	3.19	0.002*
Angle x Group [BVP]	−0.08	−0.13 to −0.04	−3.88	< 0.001*
**Random effects**	Variance	SD		
Subject (intercept)	235	15.33		
Residual	268.4	16.38		

### Path integration

**Fig 4:**
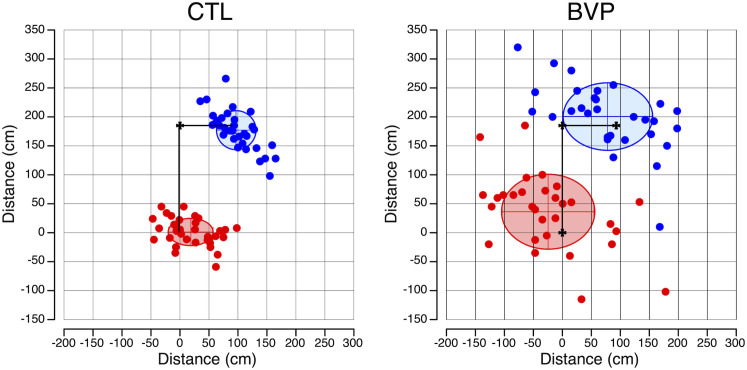
Path integration. End points for the CTL (N = 35) and the BVP (N = 30) subjects during the triangle completion task. Red symbols: trials starting with the short leg (92.5 cm); blue symbols: trials starting with the long leg (185.5 cm). The ellipses show the means and standard deviations of the coordinates of the end points for all trials starting with the short leg (blue lines and area) or the long leg (red lines and area).

**Fig 5:**
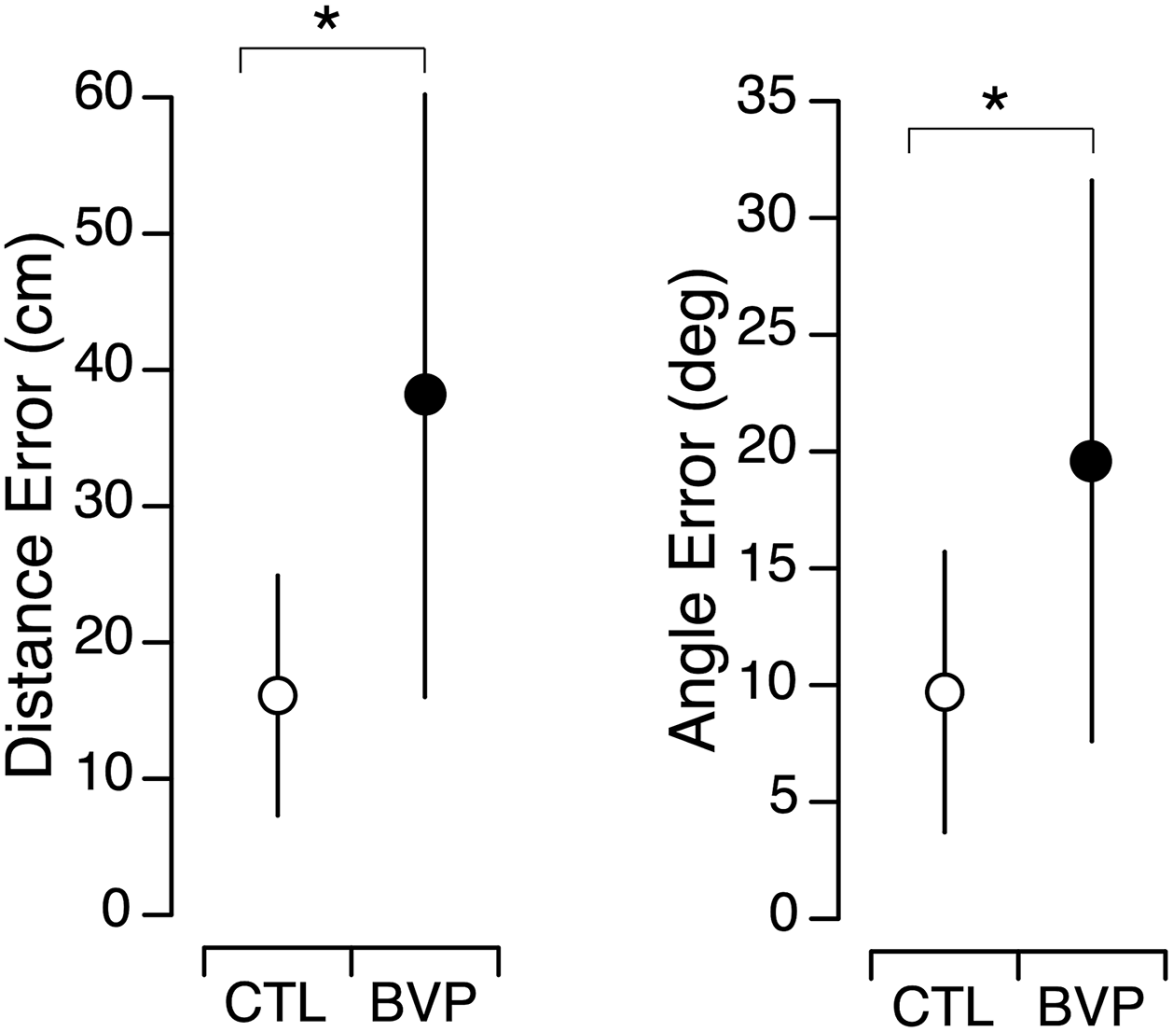
Path integration. Mean and standard deviation of absolute distance errors and angle errors during the triangle completion task in the CTL (N = 35) and BVP (N = 30) groups. * *p* < 0.05.

During the TCT, the CTL subjects positioned themselves close to the home location after both directions of rotations. In contrast, the final position of the BVP subjects showed greater variability (Fig 4). Welch’s test revealed that the BVP subjects’ perceptions of both the distance and the angle were significantly less accurate than the CTL subjects’ perceptions (Fig 5, Table 1).

The averaged distance walked by the BVP subjects was significantly longer (Welch’s t test, *p* = 0.029) at 220.5 ± 36.7 cm compared to the average distance walked by the CTL subjects (203.2 ± 18.1 cm). This result suggests that the BVP subjects demonstrate different estimation biases for goal directed straight line walking (undershoot) and for the more complex TCT navigation task (overshoot).

### Perception of duration

**Fig 6:**
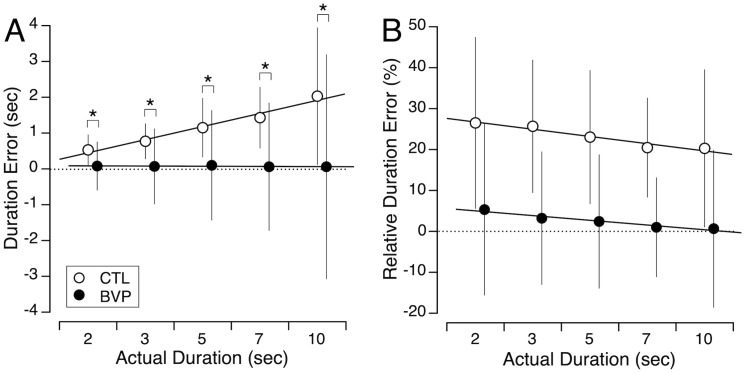
Perception of time. Mean and standard deviation of duration errors (A) and relative duration error (B) during the time reproduction task for the 5 target durations in the CTL (N = 31) and BVP (N = 29) groups. * *p* < 0.05.

The CTL subjects consistently over-reproduced the duration the square was displayed in this test, and their error increased as the target duration lengthened. Their mean relative error ranged from 23.5% to 20.3%, with an average of 23.2%. In contrast, the BVP subjects’ estimates of the square durations were remarkably accurate, with a mean relative error of 3.2% (ranging from 5.6% to 0.9%) (Fig 6). Welch’s test revealed that the differences between CTL and BVP subjects’ judgement errors were significant for all tested time intervals (Table 1).

The robust linear mixed model revealed a significant effect of target duration on judgment error (Table 4). Keeping the duration constant (at the baseline), the model showed no significant difference between the subject groups. However, a significant interaction between target duration and group indicated that the effect of target duration on judgment error was more pronounced in CTL subjects compared to BVP subjects.

**Table 4:** Robust linear mixed model comparing the differences in duration judgement error during the duration reproduction test between the CTL and BVL subject groups. For the fixed effects, the robust estimation down-weighted 19% of the residuals. For the random effects, the robust estimation down-weighted 13% of the residuals; the analysis included 296 observations in 60 subjects. * *p* < 0.05.

Fixed effects	Distance Error			
Predictors	Estimates	Confidence Interval	*t*-value	*p*-value
(Intercept)	0.21	−0.19 to 0.61	1.02	0.31
Duration	0.18	0.13 to 0.23	7.28	< 0.001*
Group [BVP]	0.07	−0.51 to 0.65	0.24	0.809
Duration x Group [BVP]	−0.25	−0.32 to −0.18	−7.04	< 0.001*
**Random effects**	Variance	SD		
Subject (intercept)	0.54	0.74		
Residual	0.73	0.86		

## Discussion

### Characteristics of the perception methods used in this study

The action of moving the body toward a target is considered the optimal method for judging egocentric distance [23]. Blind walking engages not only the perception of distance but also the memory of the target’s initial location, and involves vestibular, proprioception, motor efference copies, and spatial updating. Spatial (or imaginary) updating refers to the mental procedure the observers use to update the relative position of the target to themselves while they move with their eyes closed after they have memorized the initial position of the target. Research has shown that even after they can no longer see the target, by retaining the spatial image in their memory, subjects can more accurately determine distance than when they use other methods, such as verbal reports of distance, which require referencing units of length (e.g., meters or feet) [29]. By memorizing the location of the target, participants relied primarily on their perception of walking distance. It is possible they used their own step length as an internal reference, as walking is a familiar, everyday activity. However, blind walking is more unusual. Studies have shown that the time to walk 6 meters with eyes closed is significantly longer than when eyes were closed, especially in individuals with vestibular dysfunction [30].

Blind turning, like blind walking, relies on the participants’ memory-based representation of angle, as well as vestibular and proprioception inputs, and spatial updating as they turn in place. The TCT provides a more complex and naturalistic task because it combines both body turning and walking, closely mimicking real-life navigation. The accuracy of determining egocentric distance during the TCT depends on: (a) accurately perceiving the target’s location; (b) correctly sensing active self-motion; (c) continuously mentally updating the target’s location during movement; and (d) accurately walking toward the updated position of the target. A unique aspect of the TCT is its ability to assess spatial updating. Although blind walking, blind turning, and the TCT all involve spatial updating, the TCT combines spatial updating with trigonometric functions [31], offering a deeper evaluation of spatial navigation.

The methods used to assess perception in this study involved tasks where subjects reproduced distances (blind walking, TCT), reproduced time intervals, and produced angles (blind turning). On average, control subjects walked a distance of 1.83 meters when judging a 2-meter distance, resulting in an error of −0.17 meters. If subjects had continued walking, they believed they would have surpassed 2 meters. This relative underproduction of distance (1.83 m) compared to the actual distance (2 m) reflects a relative overestimation of the distance. This kind of dissociation is typical in action methods for measuring perception [23]. A similar interpretation applies to the performance of producing an angle during blind turning and of reproducing the duration of a display: a smaller-than-actual angle indicates an overestimation of the turn, and a longer reproduced duration compared to the actual duration suggests an underestimation of time.

### Comparison between the responses of the BVP and the CTL subjects

The CTL subjects’ overestimation of the distance during the blind walking task (where subjects stopped short) were comparable to those measured in previous studies [24,32,33]. This overestimation of distance was more pronounced for the BVP subjects than the CTL subjects. This result supports Proffitt’s theory of distance perception, which suggests that perceived distance increases as the effort required to act on a target (e.g., walking) increases [34]. According to this view, BVP subjects perceived the visual target as farther away than CTL subjects due to the greater effort needed to reach it. An alternative interpretation is that BVP subjects estimated the number of steps required to reach the target before walking and counted their steps as they moved. This estimation would be based on an internal model of step length formed when vision was available. If, after closing their eyes, the step length was reduced for safety and stability, BVP subjects would stop short of the target.

The tendency to undershoot by the vestibular patients may also reflect a cognitive strategy rather than an overestimation of movement. Individuals might prioritize safety by undershooting to reduce risks—such as stepping off a curb or into traffic—especially during distracted walking, like using a phone or talking without looking ahead.

The BVP subjects underestimated a 90-degree rotation of their bodies and overestimated a 360-degree turn. However, they accurately produced 180-degree and 270-degree turns. When asked to reproduce durations of 2–10 seconds while simultaneously reading digits, the CTL subjects underestimated these durations (waiting longer before responding), whereas the BVP subjects accurately judged the durations. The proprioceptive system, along with cognitive strategies, presumably helped the BVP subjects navigate their environment. Patients with labyrinthine defects must rely on their proprioceptive system to collect sensory information from muscles, joints, and tendons, and they use this information to compensate for their loss of vestibular function. This feedback helps maintain balance and posture when vestibular signals are impaired [35]. Proprioceptive cues enable individuals to estimate their position based on limb movement and body orientation, which is vital for maintaining stability and navigating [3]. However, proprioception provides information about the body’s position relative to itself, not the environment. Although somatosensation from the feet signals ground contact, it does not convey movement relative to an external target. The proprioceptive system also helps to map limb speed and position, allowing for real-time adjustments during dynamic activities. BVP patients often adopt compensatory strategies, becoming more visually oriented and using visual cues, when available, alongside proprioceptive feedback [17]. Moreover, integrating proprioceptive information with other sensory modalities requires higher cognitive processing because patients must consciously focus on maintaining balance and spatial orientation to offset their vestibular deficits [2].

When both distance and pointing direction tasks were combined in the TCT (walking a ~ 2 m distance, rotating the body by 116–154 deg, for a duration of ~10 s), the performance of the BVP group was less accurate and precise than the performance of the CTL group. The TCT comprehensively evaluates spatial navigation skills by requiring participants to navigate the sides of a triangle, which requires integrating distance traveled with angular orientation to return to the starting point. This dual assessment reveals how well individuals perceive and process spatial information, particularly regarding vestibular function and proprioceptive compensation. The results indicate that the BVP subjects’ performance was impaired compared to the CTL subjects when both rotations and translations were involved.

### Comparison with other studies on subjects with vestibular deficits

The results of this study, which show that BVP patients performed less accurately and precisely than the control group during the TCT involving both distance and pointing direction, both align with and contrast with previous studies. Similar to earlier research, we found that BVP patients show impaired path integration abilities during the TCT compared to healthy individuals [36,37]. However, Glasauer et al. [15] reported that labyrinthine deficient subjects could accurately perceive distance during blind walking along a triangular path, but struggled to navigate turns effectively. The discrepancy between Glasauer et al. [15] findings and the current study may be due to differences in the nature of the vestibular deficits among participants. Glasauer’s subjects had heterogeneous vestibular impairments, including unilateral deficits or damage caused by neurectomy or toxicity, which might explain the more limited difficulties they experienced compared to the consistent impairments observed in the BVP group in our study. Additionally, our study highlights the significance of integrating both distance and angular orientation, demonstrating that BVP patients’ impaired vestibular function affects their ability to process both types of spatial information, unlike in the previous study where only rotation was affected.

In most cases, bilateral vestibulopathy primarily involves deficits in the peripheral vestibular system, leading to alterations in the central processing of signals from the vestibular, proprioceptive, and visual systems [18]. This is supported by observed changes in cognitive tests that assess visuospatial ability, executive function, perceptual motor speed, and episodic memory in the BVP patients [38–41]. Additionally, BVP subjects exhibited performance impairments during a timed-up-and-go task with their eyes open, impairments that are comparable to those observed in astronauts immediately after they return from long-duration spaceflight [16]. Exposure to microgravity simulates loss of vestibular otolith function during head tilts, which recovers within a few days after returning to Earth [7]. It has been shown that astronauts have an altered perception of distance during spaceflight [8], and angular errors were recorded when astronauts walked along a triangular path within a few hours of returning from a 2-week spaceflight [9]. Interestingly, astronauts demonstrate greater accuracy in reproducing time intervals ranging from 2 seconds to 1 minute during spaceflight compared to on Earth. Their performance in space is comparable to that of the BVP subjects observed in this study [5]. According to the internal clock model [42], it has been hypothesized that this increased accuracy may result from an acceleration of the internal clock, driven by changes in vestibular inputs within the weightless environment [10].

## Conclusion

The novelty of this study lies in its detailed exploration of how individuals with bilateral vestibulopathy perceive distance, angle, and time, both separately and in combination. The study reveals that while BVP patients can compensate for their vestibular deficits in simpler tasks by relying on alternative sensory systems like proprioception and vision, this compensation becomes less effective when tasks require the integration of multiple spatial and temporal cues, such as in the triangle completion task. Additionally, the study finds that BVP patients demonstrate accurate time perception, unlike healthy controls, challenging previous assumptions about the role of the vestibular system in time perception. The use diagnostic criteria consensus and a larger sample size helps fill gaps in prior research, offering a more comprehensive view of the adaptive strategies used by individuals with vestibular impairments. These insights broaden the understanding of vestibular disorders and spatial perception, highlighting how deficits in vestibular function impact the integration of spatial and temporal information and affect everyday navigation and coordination. Furthermore, the findings are particularly relevant to space exploration, where microgravity simulates vestibular dysfunction, potentially informing strategies to address perceptual challenges in astronauts.

## Limitations of the study and future recommendations

Tests of vestibular reflexes were conducted solely in the BVP subjects and not in the CTL group. However, CTL subjects were excluded from the study if they had current or past vestibular disorders, suggesting the vestibular function of the CTL subjects selected for this study likely fell within the normal range.

The presence of safety operators during the tests could have influenced the BVP subjects’ responses in several ways. Firstly, the operators may have provided a sense of security that reduced anxiety and allowed the BVP subjects to focus more on the task at hand. This support might have led to more accurate performance in distance judgment and balance. However, the operators’ physical assistance or guidance could also have created a reliance on external support, potentially masking the BVP subjects’ true navigational abilities and influencing their natural movement patterns.

During the blind turning test, some BVP subjects paused after each 90-degree rotation of their body. This strategy of turning in structured increments may have helped them orient themselves more easily, reducing cognitive load and allowing them to focus on the mechanics of the task. However, it may also have led to less accurate representations of their natural turning abilities because it does not capture the fluidity of movement typically used in real-life situations.

The age disparity between the BVP and the CTL groups may account for some of the differences observed between study groups. Vestibular function diminishes with age [43,44], and older adults with vestibulopathy perform poorer on dynamic spatial navigation tasks than healthy younger and older adults [16,37].

Future studies should consider measuring walking time with eyes open during both the distance perception task and TCT; characterizing blind turning strategies in the BVP group (e.g., segmented vs. continuous turns); testing the TCT with equal-amplitude rotations in both directions; and including age-matched groups to allow inclusion of age as a covariate in the analysis.

## Supporting information

S1 Text Table supplementary. (TXT)
